# A New Islet Transplantation Method Combining Mesenchymal Stem Cells with Recombinant Peptide Pieces, Microencapsulated Islets, and Mesh Bags

**DOI:** 10.3390/biomedicines8090299

**Published:** 2020-08-21

**Authors:** Ryo Kogawa, Kentaro Nakamura, Yusuke Mochizuki

**Affiliations:** Bioscience and Engineering Laboratory, Research and Development Headquarters, Fujifilm Corporation, Kanagawa 258-8577, Japan; kentaro.a.nakamura@fujifilm.com (K.N.); yusuke.mochizuki@fujifilm.com (Y.M.)

**Keywords:** islet transplantation, CellSaic, microencapsulation, MSC, diabetes

## Abstract

Microencapsulated islet transplantation was widely studied as a promising treatment for type 1 diabetes mellitus. However, micro-encapsulated islet transplantation has the following problems—early dysfunction of the islets due to the inflammatory reaction at the transplantation site, and hyponutrition and hypoxia due to a lack of blood vessels around the transplantation site, and difficulty in removal of the islets. On the other hand, we proposed a cell transplantation technique called CellSaic, which was reported to enhance the vascular induction effect of mesenchymal stem cells (MSCs) in CellSaic form, and to enhance the effect of islet transplantation through co-transplantation. Therefore, we performed islet transplantation in diabetic mice by combining three components—microencapsulated islets, MSC-CellSaic, and a mesh bag that encapsulates them and enables their removal. Mesh pockets were implanted in the peritoneal cavity of Balb/c mice as implantation sites. After 4 weeks of implantation, a pocket was opened and transplanted with (1) pancreatic islets, (2) microencapsulated islets, and (3) microencapsulated islets + MSC-CellSaic. Four weeks of observation of blood glucose levels showed that the MSC-CellSaic co-transplant group showed a marked decrease in blood glucose levels, compared to the other groups. A three-component configuration of microcapsules, MSC-CellSaic, and mesh bag was shown to enhance the efficacy of islet transplantation.

## 1. Introduction

Diabetes is characterized by chronic hyperglycemia caused by abnormalities in insulin secretion, action, or both. Eighty to ninety percent of cases of diabetes in children and adolescents suffer from type 1 diabetes resulting from complete autoimmune destruction of pancreatic β-cells, through cellular immune responses [1].

Islet transplantation is a promising treatment for type 1 diabetes. However, pancreatic islet transplantation poses several problems. These include destruction by autoimmunity, allogeneic, or xenobiological rejection, limited supply and suboptimal yields of islet procurement and isolation, hypotrophy and hypoxia of transplanted islets, and exposure to the toxic effects of immunosuppressive agents [2]. These are thought to contribute to early graft failure [3]. In addition, there is the risk of not being able to get it out if something goes wrong [4].

One of the approaches to solve these problems is the encapsulation of pancreatic islets [5]. This technology aims to encapsulate therapeutic cells, such as pancreatic islets, within biocompatible materials, with the objective of providing a support structure to the islets that replicates the native islet micro- and macro-environment, and offers immunoisolation once implanted [2]. Therefore, microencapsulation prior to implantation might address some of these issues. (i) Overcome the shortage of human donors due to the potential for transplantation of heterologous pancreatic islets and other insulin-producing cell phenotypes. (ii) Provide a delimited structured scaffold that prevents loss of islets after transplantation. (iii) Eliminate the need for immunosuppression [2,5,6].

However, microencapsulated pancreatic islets still have challenges in stable insulin withdrawal and long-term functional maintenance. The causes are early dysfunction due to the inflammatory response around the transplantation site, and poor implantation due to the lack of blood vessels around the transplantation site and hypotrophic and hypoxic conditions [7,8].

On the other hand, we reported a cell transplantation platform called “CellSaic”, which enhances cell viability. “CellSaic” is a term coined from “Cell” and “Mosaic”. CellSaic is a three-dimensional structure made by combining cells with a new bioresorbable material, a recombinant protein for medical use (RCP). RCP is a recombinant protein produced by the yeast Pichia pastoris and differs from conventional animal collagen as there is no risk of infection, such as bovine spongiform encephalopathy [9,10]. Unlike spheroids, the spaces between the petaloid RCP pieces of the CellSaic platform lead to greater penetration of substances into the cells, which prevents cell death [11]. MSCs have revascularization and immunomodulatory functions [12], and are known to enhance islet function when co-transplanted with pancreatic islets [13,14]. We also reported that co-transplantation of MSC-CellSaic with islets is even more effective than simply co-transplanting MSC with islets [11]. Therefore, MSC-CellSaic could be an approach to solve problems such as vascular deficiency around microencapsulated pancreatic islets and rejection caused by inflammation.

In addition, macrocapsules are the solution to the problem of microcapsules that cannot be used to remove pancreatic islets. Macrocapsules, like microcapsules, are widely studied as a method of islet transplantation [15]. However, there are challenges, such as necrosis of pancreatic islets present in the center of the device, associated with poor nutrient penetration into the interior, due to the size of the device and a lack of blood vessels [16]. We expect that a combination of microcapsule and macrocapsule elements will enable microcapsule islet transplantation, which can be excised. Since the immune isolation function is also carried out by the microcapsules, the macrocapsules can be made into a mesh-bag-like device with a large diameter that can be passed through cells and blood vessels, to provide nutrients to the interior. In addition, the macrodevice can be embedded in the recipient’s transplant site prior to islet transplantation, to create pockets where the islets, whose foreign substance reaction has subsided can easily become engrafted [17].

In the present study, we investigated the combination of MSC-CellSaic, encapsulated islets and macro pockets in the hope that the problem of encapsulated pancreatic islets could be solved.

## 2. Experimental Section

### 2.1. Animals

All animals were purchased from Charles River, Japan (Yokohama, Japan); MSCs were isolated from C57BL6/N (6 weeks old, ♂). Pancreatic islets were isolated from Wistar rats (7–8 weeks old, ♂). Balb/c (6–7 weeks old, ♂) was used as the recipient for islet transplantation. The C57BL/6N (6–7 weeks old, ♂) was used to assess angiogenicity. The experimental protocol was approved by the animal control committee of FUJIFILM Corporation and all procedures were carried out (A-1-180424 (26 September 2018), A-1-180448 (28 September 2018)).

### 2.2. Materials

MSCs were generated as previously described [18]. In brief, adipose tissue from a C57BL/6N was cut into small pieces and digested at 37 °C for 1 h, with 2 mg/mL collagenase (Nitta gelatin, Tokyo, Japan) in Dulbecco’s phosphate-buffered saline. The sample was centrifuged at 600× *g* for 5 min, washed, centrifuged again, and seeded into a flask containing MSCGM (PT-2501, Lonza, Basel, Switzerland). The medium used for MSC was high-glucose DMEM (043-30085, Wako, Osaka, Japan) containing 10% fetal bovine serum (30-2020, ATCC, Manassas, VA, USA), and penicillin streptomycin/amphotericin B (161-23181, Fujifilm Wako Pure Chemical, Osaka, Japan). MSCs were negative for the endothelial markers 49e and CD117 and for the hematopoietic markers CD34, CD45, and CD11b. These cells expressed Sca-1, CD44, CD29, and CD90 [18]. RCP pieces were generated as previously described, and we used the petaloid pieces [10].

### 2.3. Islet Isolation

Islets were isolated from rat pancreas using 1 mg/mL collagenase V (Sigma type V; Sigma Chemicals, St Louis, MO, USA) solution in 10 mL HBSS, through a common bile duct catheter. After the spleen, the duodenum and stomach were removed, and the pancreas was digested (30 min, 37 °C). Thereafter, density gradient centrifugation was performed, as previously reported [19,20]. Islet survival was evaluated by the ADP/ATP ratio assay, using the ADP/ATP Ratio Assay Kit, ApoSENSOR (K255-200, BioVision, San Francisco, CA, USA).

### 2.4. Preparation of an Animal Model of Diabetes

One week prior to transplantation, a single intraperitoneal injection of 250 mg/kg body weight streptozotocin (Fujifilm Wako Pure Chemical, Osaka, Japan) into mice made them diabetic; mice with non-fasting blood glucose levels above 300 mg/dL for two consecutive days, were recognized as diabetic individuals.

### 2.5. Preparing a Transplant Pocket

A 10 mm × 10 mm × 2 mm silicon plate was placed in a 15 mm × 15 mm nylon mesh bag and the enclosure was sealed with a heat sealer. The skin and muscles of the abdomen of the recipient mice were incised 1.5~2.5 cm and fixed by sewing to the muscles inside the abdominal cavity.

### 2.6. Preparing Alginate Microcapsules

Alginate microcapsules were prepared using the Encapsulator B-390 (BUCHI, Flawil, Switzerland). Encapsulation was performed as previously reported [21,22]; pancreatic islets were suspended in 1.8% sodium alginate solution (BUCHI, Flawil, Switzerland) and encapsulated through an encapsulation machine. The alginate beads were cross-linked using 100 mM CaCl_2_. The capsules were coated with 0.05% poly-l-lysine (PLL) (Fujifilm Wako Pure Chemical, Osaka, Japan; MW 15,000–30,000). The dilute sodium alginate (0.03%) was added to cover the PLL.

### 2.7. In Vitro Evaluation of Microencapsulated Pancreatic Islets

The insulin release performance of the prepared microencapsulated and unencapsulated pancreatic islets was evaluated in terms of their glucose responsiveness. Ten to twenty capsules and islets were picked up and washed twice with medium for rat islets containing 3 mM glucose (Cosmo bio, Tokyo, Japan). Capsules and islets were incubated at 37 °C for 1 h, in a low concentration glucose solution (3 mM). They were then transferred to a high concentration glucose solution (20 mM) and incubated at 37 °C for 1 h in the same way. Each medium was collected and insulin levels were measured with a rat Insulin ELISA kit (ALPCO Diagnostics, Salem, NH, USA).

### 2.8. Evaluation of Antibody Permeability of Microcapsules

A biological isotonic solution was prepared by weighing potassium chloride (4.1 g), sodium chloride (8.0 g), calcium chloride (0.3 g), magnesium chloride, and 6-hydrate (0.46 g), and mixing it with 1000 mL of water for injection and 500 mL of PBS buffer pH 7.4 (10010-023, Thermo Fisher Scientific, Waltham, MA, USA). After mixing, it was sterilely filtered through a bottle top filter (Stricup). A total of 3 μL of 1 μg/μL Mouse IgG Isotype Control Alexa Fluor 488 Conjugated (bs-0296P-A488, Funakoshi, Tokyo, Japan) was added to 897 μL of biological isotonic solution and diluted. The mixture was shaded with aluminum foil. A microcapsule containing Goat anti-Mouse IgG-bound Dynabeads (Invitrogen, Carlsbad, CA, USA) was added to a 96-hole glass bottom plate with 50 μL of biological isotonic solution. Then, 2.5 μL of fluorescent antibody diluent was added. The time dependence of the internal fluorescence intensity was observed under a fluorescence microscope (Keyence BZ-X710, magnification: 10×, GFP filter, reduced fading mode, exposure time 1 s).

### 2.9. Formation of MSC CellSaic Platforms

MSC were cultured at 37 °C in a 5% CO_2_ humidified atmosphere. CellSaic platforms, containing MSC, were prepared by mixing MSC (1.2 × 10^6^ cells/mL) and RCP pieces (1 mg/mL) in MSC medium; this mixture was seeded on a 35-mm dish of EZ SPHERE 4000-903SP (AGC Techno Glass, Haibara, Japan). MSC-Spheroid was prepared in the same way, except for the RCP pieces (Figure S1).

### 2.10. Transplantation of Microencapsulated Pancreatic Islets

Mice were transplanted under isoflurane anesthesia. Recipients were divided into three groups—(i) islet group (*n* = 5; 1000 islets alone); (ii) encapsulated islet group (*n* = 5; microencapsulated islets 1000 alone); and (iii) CellSaic group [*n* = 5; encapsulated islets 1000 and MSC-CellSaic (total: 1.2 × 10^6^ cells and 1 mg)]. The abdomen of a diabetic mouse was incised and the mesh bag inside was checked. The mesh bag was cut open on one side and the silicone plate inside was removed. Islets, encapsulated islets, or encapsulated islets and MSC-CellSaic were placed in the space after extraction and then sealed. For one month after transplantation, blood glucose levels were measured, except for holidays.

### 2.11. Comparison of Angiogenesis Induction Performance

MSC-Spheroid or MSC-CellSaic was enclosed in bags made of polysulfone membranes that did not pass through the cells, and were implanted subcutaneously in mice. The number of cells was 1.2 × 10^6^ cells per animal in both cases. After 2 weeks of transplantation, bags of polysulfone were removed as muscle and skin together, and tissue specimens were made.

### 2.12. Histological Evaluation

In a diabetic model in which the islets were transplanted, pockets were removed from the peritoneal cavity 28 days after transplantation, and fixed in a 10% phosphate-buffered formalin solution. H&E-stained sections were prepared for histological examination. For immunostaining, deparaffinized sections were first incubated with anti-insulin antibodies (I2018; Sigma-Aldrich, St. Louis, MO, USA) and then with LSAB2 biotin-conjugated secondary antibodies (K1015; Dako, Glostrup, Denmark) plus streptavidin-horseradish peroxidase (K1016; Dako, Glostrup, Denmark), and the signals were developed using diaminobenzidine solution (K3468; Dako, Glostrup, Denmark). Next, sections were counterstained with hematoxylin [11]. In the angiogenesis assessment model, pockets were removed from the abdominal cavity at 14 days after transplantation and fixed in a 10% phosphate-buffered formalin solution. H&E-stained sections were prepared for histological examination

### 2.13. Statistical Analysis

Results are presented as mean ± standard deviation. All comparisons of results were analyzed with Student’s *t*-test; *p* < 0.05 was considered to be statistically significant.

## 3. Results

### 3.1. In Vitro Characterization of Encapsulated Pancreatic Islets

**Figure 1:**
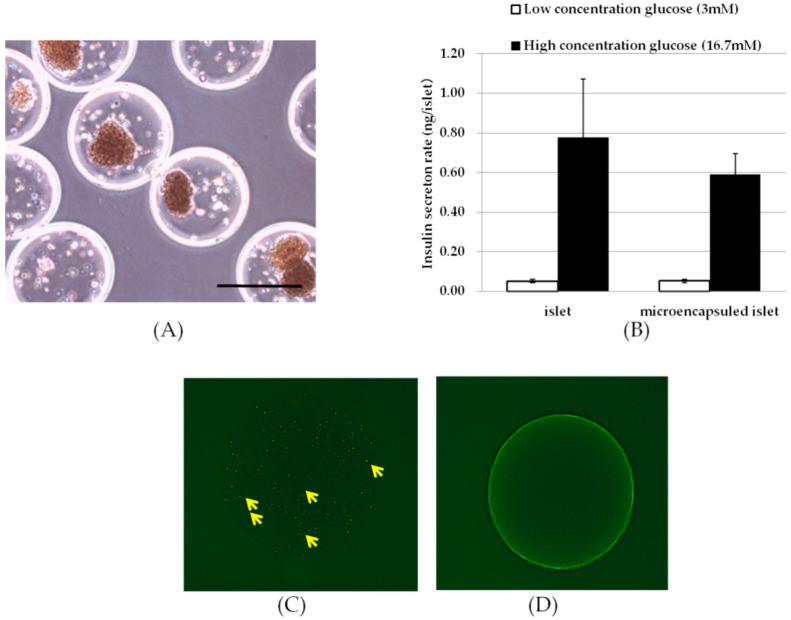
Photographs and in vitro performance of the microencapsulated islets. The capsules contain 0–4 islets per capsule, and the diameter of the capsule is approximately 600 μm (**A**). Microencapsulated and unencapsulated pancreatic islets were stimulated in low concentration glucose solution (white bar) for 1 h, followed by incubation in high concentration glucose solution (black bar) for 1 h, and the amount of insulin released was evaluated (**B**). Mouse IgG permeability to capsules enclosed with DYNA beads immobilized with anti-mouse IgG was assessed; image after immersion of an unPLL-coated alginate capsule in a solution containing fluorescently labeled mouse IgG for 10 min (**C**). Yellow arrows indicate invaded fluorescently labeled mouse IgG. image of PLL-coated alginate capsules after 60 min of immersion in a solution containing fluorescently labeled mouse IgG (**D**); Scale bars = 500 μm.

To evaluate the combined effect of MSC-CellSaic and encapsulated islets, we first prepared encapsulated islets, each of which contained 0~4 islets (Figure 1A).

The capsules were clean and spherical, without distortion. The prepared encapsulated pancreatic islets were evaluated in a glucose responsiveness test to confirm that they were functional (Figure 1B). The ADP/ATP ratio assay also confirmed the survival of the islets (Figure S2). The insulin permeability of unencapsulated islets was 0.052 ± 0.008 ng/islet in low glucose medium and 0.779 ± 0.293 ng/islet in high glucose medium, with a stimulation index (SI) of 15.8 ± 7.5. Encapsulated islets had an insulin release of 0.050 ± 0.009 ng/islet in low glucose, 0.589 ± 0.106 ng/islet in high glucose and 11.3 ± 1.3 for SI. Encapsulated pancreatic islets showed lower insulin release than unencapsulated islets, but the SI was greater than 2 and showed adequate glucose responsiveness. The blocking properties of immune molecules were assessed by observing the accumulation of fluorescein-modified mouse IgG that permeated from outside the capsule onto DYNA beads in the microcapsules. The blocking performance of antibodies might not need to be as high, and it was reported that heterologous pancreatic islets with microcapsules that are simply cross-linked with barium alginate have sustained effects in the abdominal cavity for more than three months [23,24]. Therefore, the target performance of the antibody blocking was set to be more than barium cross-linked alginate. In the barium alginate capsule prepared as a control, fluorescence was observed inside the capsule, 30 min after the addition of fluorescent-modified mouse IgG. In contrast, PLL-coated capsules did not show any fluorescence even after 60 min. In addition, fluorescent antibodies were detected after 10 min in the uncoated microcapsules (Figure 1C,D).

### 3.2. Effect of MSC-CellSaic on Blood Glucose Control

**Figure 2:**
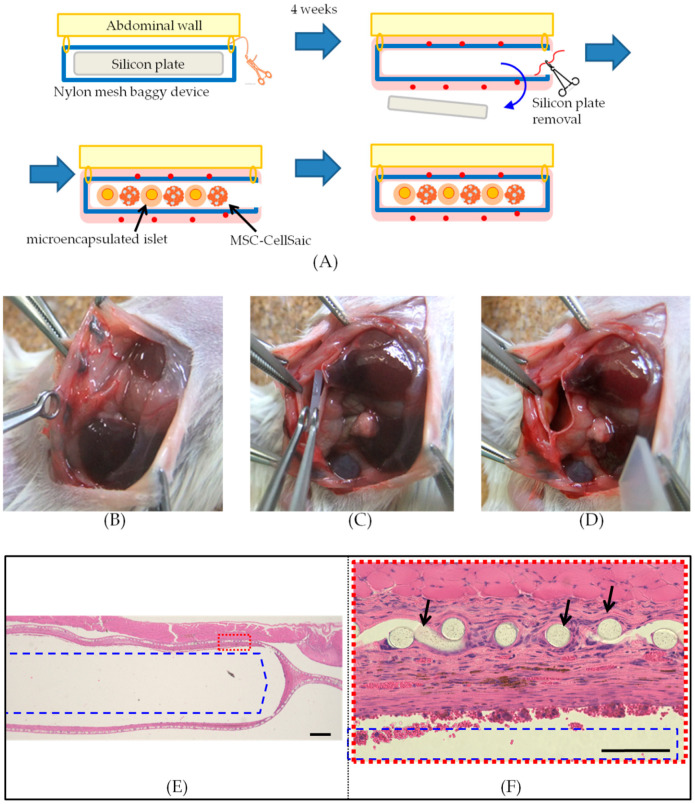
A method for making nylon mesh pockets for implantation. Illustration of the procedure of transplanting islets into a pocket created by placing a nylon mesh bag in the peritoneal cavity of a Balb/c mouse (**A**). A nylon mesh bag with a silicon plate enclosed in the abdominal wall was sewn in. Four weeks after fixation of the mesh device, after the tissue reaction subsided and blood vessels formed, the silicone plate was removed (**B**–**D**). The transplant was finished by placing the graft in the space, after the silicone plate was removed. Tissue sections, 4 weeks after a nylon mesh device enclosing a silicone plate was sewn into the intra-abdominal wall (**E**,**F**). The blue rectangle shows the space in which the silicon was encased. The black arrow shows the nylon mesh. The red square indicates the region of (**F**). Scale bar = 500 µm (E), 100 µm (F).

Pockets for co-implantation of encapsulated pancreatic islets and CellSaic were created in the peritoneal cavity of mice (Figure 2A).

A nylon mesh bag filled with silicone was fixed in the abdominal cavity and the internal silicone was removed 4 weeks later. A membrane of fibers was formed around the fabricated pocket, but there was a cavity inside where the silicone was present (Figure 2B–F).

**Figure 3:**
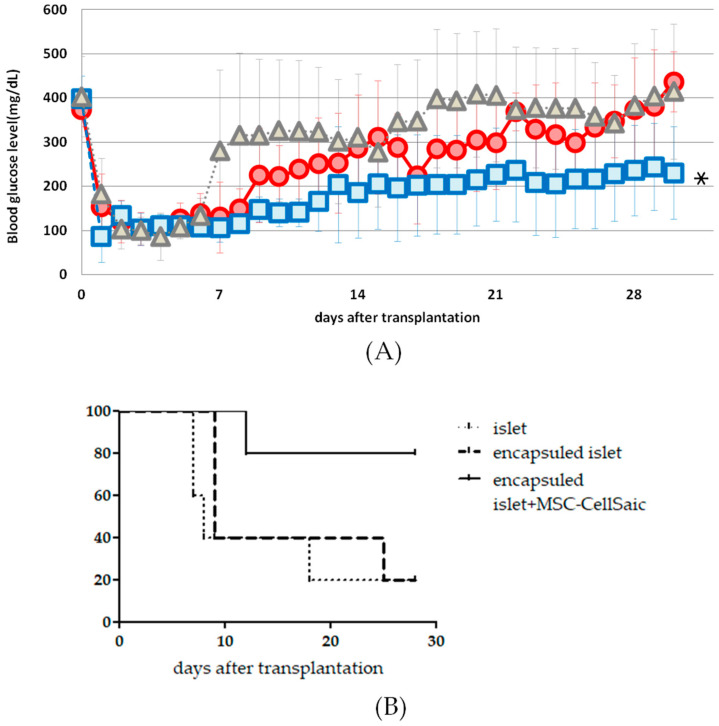
Changes in blood glucose levels in Balb/c mice under non-fasting conditions after islet transplantation (**A**).Transplantation was performed on DAY0 and continued until 28 days later, to measure blood glucose levels (*n* = 5). □: Encapsulated islet + MSC-CellSaic group O: Encapsulated islet group Δ: Islet group. Recording of changes over time in the rate of normalization of blood glucose levels after transplantation (**B**). If the blood glucose level exceeded 250 mg/dL on two consecutive occasions, the mice were considered hyperglycemic and dropped out; * *p* < 0.05. We compared the Encapsulated islet + MSC-CellSaic group with the Encapsulated islet and Islet groups, using a one-way analysis of variance with *T* test for blood glucose levels, after 4 weeks of transplantation.

Inside the fabricated pockets, non-encapsulated pancreatic islets, encapsulated islets, encapsulated islets and MSC-CellSaic, were implanted. One month after transplantation, the blood glucose levels of the transplanted mice were measured (Figure 3A).

At 1 month after transplantation, the mean blood glucose level of the non-encapsulated islets was 414.5 ± 153.2 mg/dL and 436.8 ± 68 mg/dL for the encapsulated islets and 230.2 ± 104.9 mg/dL for the encapsulated islets and MSC-CellSaic, and the encapsulated islets and the MSC-CellSaic groups significantly reduced the blood glucose levels. At 1 month post-transplantation, the mean blood glucose control rate for non-encapsulated islets was 20%, 20% for encapsulated islets, and 80% for encapsulated islets and the MSC-CellSaic, which also suggested that CellSaic co-transplantation enhanced islet viability (Figure 3B).

### 3.3. Histological Observations

**Figure 4:**
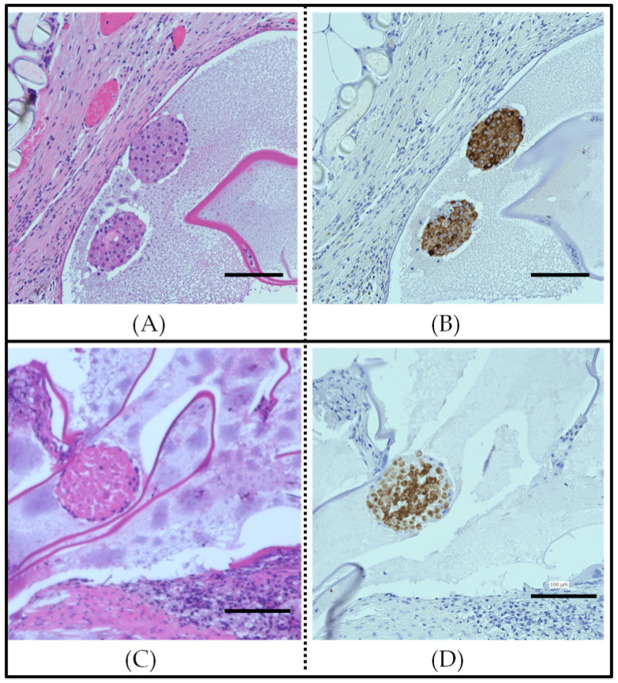
Histological analysis of the interior of a mesh device that was removed one month after implantation. Hematoxylin & eosin staining (**A**) and tissue staining with anti-insulin antibody (**B**) inside the mesh device extracted from the encapsulated pancreatic islets + MSC-CellSaic group. Hematoxylin & eosin staining (**C**) and tissue staining with anti-insulin antibodies (**D**) inside the mesh device extracted from the encapsulated islet group. Insulin positive areas are shown in gray. Scale bar = 100 µm.

Tissue specimen observations of the grafts were made. One month after transplantation, pockets containing capsules were removed and subjected to insulin immunostaining and hematoxylin & eosin (H&E) staining (Figure 4).

In the encapsulated islets and MSC-CellSaic transplantation group, insulin-positive areas were scattered and live islets with stained nuclei were identified (Figure 4A,B). On the other hand, in the encapsulated islets, few insulin-positive areas were found, and many islets were found to have died in the center of the islets due to necrosis. It was also observed that inflammatory cells were densely packed around the capsule in the encapsulated islet transplant group (Figure 4C,D). These results indicate that MSC-CellSaic inhibits the inflammatory response induced by the islet of the capsule.

The area and number of blood vessels in the pericapillary pocket of the micro-encapsulated islet transplant group and the micro-encapsulated islet and CellSaic co-transplant groups were measured and compared from tissue specimens.

**Figure 5:**
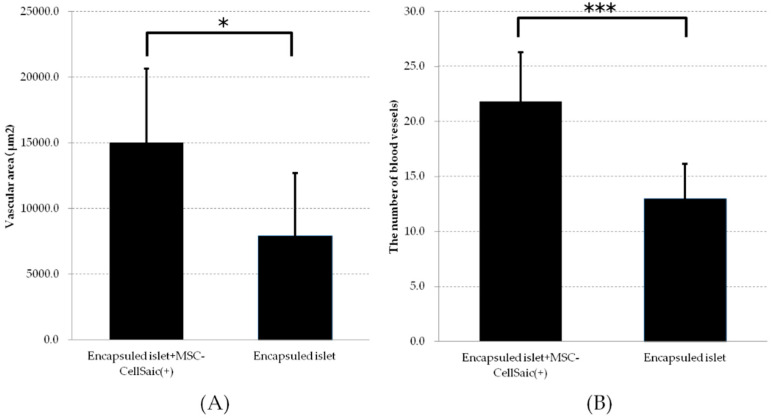
Image analysis of blood vessels around the capsule in a mesh device. From hematoxylin and eosin-stained images of sections of mesh devices from the encapsulated pancreatic islets + MSC-CellSaic group and the encapsulated islet group, the area of blood vessels near the capsule (**A**) and (**B**) the number of blood vessels observed in the field of view were measured. (*n* = 5), * *p* < 0.05, *** *p* < 0.005.

The area of the CellSaic co-transplant group was 15025.3 ± 5617.3 µm^2^ and the number of blood vessels was 21.8 ± 4.5. The vessel area in the encapsulated islet transplant group was 7904.3 ± 4803.9 µm^2^ and the number of vessels was 13.0 ± 3.2. Therefore, we found that MSC-CellSaic co-transplantation might enhance pericapsule angiogenesis and contribute to the survival of pancreatic islets (Figure 5A,B).

### 3.4. Histological Comparison of Angiogenesis Induction

**Figure 6:**
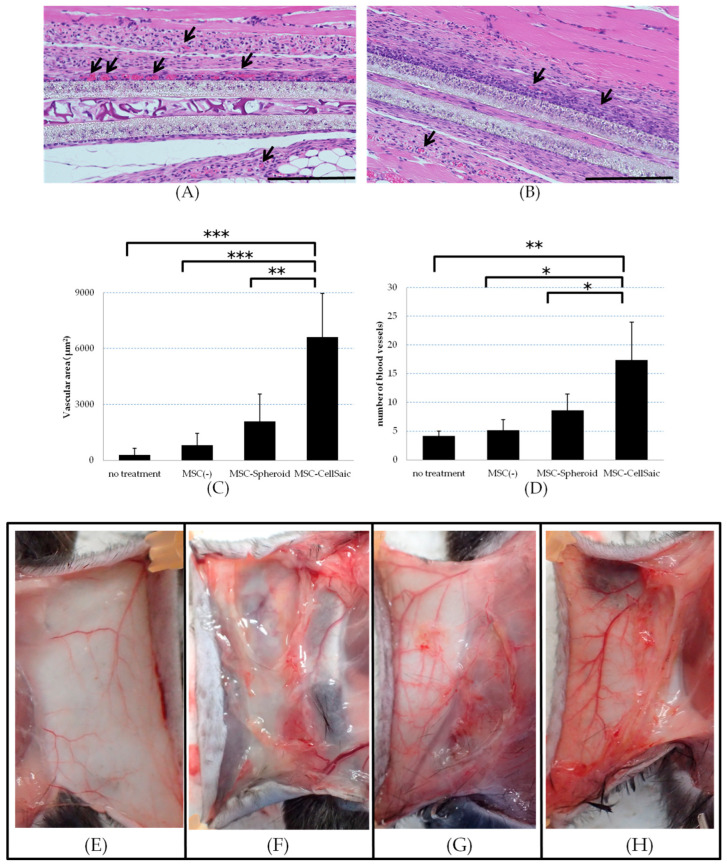
Evaluation of the angiogenicity of MSC-CellSaic. Embedding MSC-CellSaic or MSC-Spheroid in a cell-impermeable polysulfone bag under the dorsal skin of C57BL/6N mice. Two weeks after transplantation, the grafts were removed along with the skin and muscle, and tissue specimens were prepared, and hematoxylin & eosin staining was performed in the MSC-CellSaic (**A**) and MSC-Spheroid (**B**) groups. Tissue specimens were imaged and the area (**C**) and number of vessels (**D**) in the field of view within 100 μm of the vicinity of the polysulfone membrane were analyzed. No treatment indicates an individual with no subcutaneous transplantation, and MSC(-) indicates an individual with a polysulfone bag implanted with no MSCs enclosed. Subcutaneous dorsal (**E**) of no treatment C57BL/6N. Dorsal subcutaneous photographs of C57BL/6N after extraction of empty polysulfone bag (**F**), MSC-Spheroid (**G**), and MSC-CellSaic (**H**). (*n* = 5) Scale bar = 200 μm, * *p* < 0.05, ** *p* < 0.01, *** *p* < 0.005.

We investigated the potentiation of angiogenesis-inducibility of MSC in CellSaic morphology. MSC-CellSaic or MSC-Spheroid was implanted in a fine polysulfone bag that is impermeable to the cells, into the dorsal skin of mice. Two weeks after transplantation, the bags were removed along with the skin and the muscle, and tissue specimens were made. The prepared tissue specimens were stained with hematoxylin and eosin. Tissue specimens were observed and the total area and number of vessels present around the membrane were measured and compared. Tissue specimen observations showed a large number of vessels in the MSC-CellSaic group that were clearly in the immediate vicinity of the membrane (Figure 6A,B).

The results showed that the area of vessels in the CellSaic group within 100 µm of the membrane was 6610.2 ± 2335.8 µm^2^ and the number of vessels was 17.4 ± 6.6. The area of vessels in the Spheroid group within 100 µm of the membrane was 2090.6 ± 1481.3 µm^2^, and the number of vessels was 8.6 ± 2.9 (Figure 6C,D). The dorsal subcutis was observed and compared after the removal of polysulfone bags in each group, 2 weeks after transplantation (Figure 6E–H). More blood vessels were observed in the subcutaneous area of the mice implanted with the MSC-filled polysulfone bags, compared to the untreated or empty bags. In addition, the MSC-CellSaic group showed more redness throughout the subcutaneous area than the MSC-Spheroid group, which was predicted to be more vascular. The results showed that the CellSaic enhanced the angiogenesis-inducing function of MSCs. This function was confirmed through the non-cellular membrane, which was related to the cytokines released by the MSC.

## 4. Discussion

MSC-CellSaic enhances the function of microencapsulated pancreatic islets by inducing angiogenesis and suppressing the inflammatory response. It was previously reported that the anti-inflammatory effect of MSC-CellSaic was enhanced over that of non-CellSaic MSCs [25]. Making MSCs CellSaic secretes more of the anti-inflammatory cytokine TSG-6, which is an important cytokine that exerts its effects in various inflammatory models [26]. In in vitro experiments, the release of TSG-6 in the CellSaic form was up to 3.1-fold higher than in the non-CellSaic form [25]. In this study, the weakened inflammatory response in the MSC-CellSaic co-transplant group might be due to this increased TSG-6 release. In experiments in which microencapsulated pancreatic islets and MSC-CellSaic were subcutaneously transplanted into the skin of mice, the inflammatory response around the capsule was calm in the early stages of transplantation, even within 14 days of transplantation (Figure S3). These results suggest that MSC-CellSaic is capable of suppressing inflammation and immune rejection from early transplantation, up to 4 weeks, and longer-lasting effects can be expected if the MSC-CellSaic is still present.

It was also reported that MSC-CellSaic has superior vascular induction into the interior of the cell mass than the MSC-Spheroid [9]. In the present study, the induction of angiogenesis around the non-cellular membrane was thought to be due to the increased secretion of VEGF and HGF, factors involved in vascular induction, as well as TSG-6. In fact, it was been reported that MSC-CellSaic increased the release of various cytokines and growth factors, such as IL-8, ENA-78, bFGF, and VEGF [10]. Other functions of MSCs that contribute to islet viability included inhibition of apoptosis, scaffolding [27], and protection from cytotoxicity [28,29,30,31,32]. Other than angiogenesis and inhibition of inflammation, the performance of MSCs in the form of CellSaic might also be altered to influence the current study.

In this case, each islet was encapsulated in a mesh bag to place the encapsulated islets and CellSaic near each other, but it was expected to become a more effective islet transplantation method by changing the number, size, and placement of CellSaic. Especially if the effect of MSC was largely due to cytokines, the localization of CellSaic should be closer to the islets. In this respect, CellSaic had the advantage of controlling localization better than a single cell, which is easily dispersed. It was also reported that in islet transplantation, prior vascular induction at the transplant site improved islet implantation and weakened immune rejection [33,34,35]. In the present study, only silicone plates were enclosed when creating pockets with mesh bags. However, even then, the insertion of MSC-CellSaic might increase the success rate of islet transplantation as a larger number of blood vessels can be induced in and around the pocket before transplantation. Due to its many functions, MSC has a very high affinity for islet transplantation. It is hoped that islet transplantation will be further improved by better handling of MSC-CellSaic, which enhances the effectiveness of MSC.

## 5. Conclusions

The combination of MSC-CellSaic and microencapsulation and pockets made with mesh bags was found to be effective in the survival of pancreatic islets.

## Supplementary Materials

The following are available online at https://www.mdpi.com/2227-9059/8/9/299/s1.
