# Supplementary material for: A New Islet Transplantation Method Combining Mesenchymal Stem Cells with Recombinant Peptide Pieces, Microencapsulated Islets, and Mesh Bags
Source: Biomedicines. 2020 Aug 21;8(9):299. doi: 10.3390/biomedicines8090299 (PMC7555598; doi:10.3390/biomedicines8090299)
Supplement: Supplementary file 1 [file biomedicines-08-00299-s001.pdf]

biomedicines

Supplementary Materials

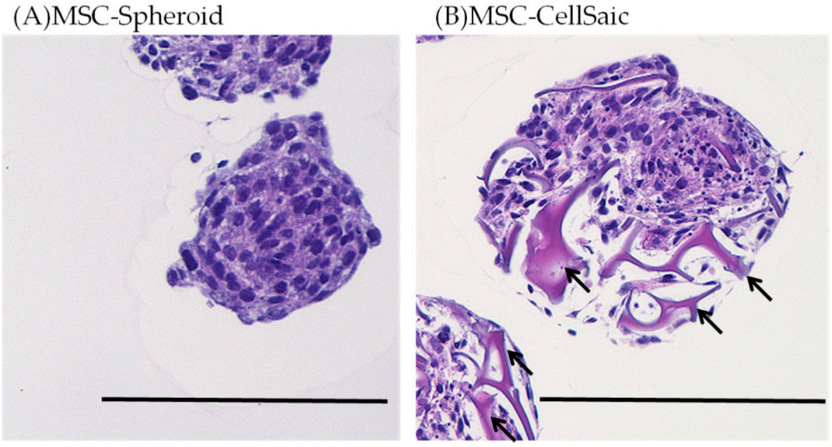

Figure S1. Histology images of MSC-Spheroid and MSC-CellSaic. Hematoxylin and eosin stained images of MSC-Spheroid (A) and MSC-CellSaic before transplantation. Arrows indicate recombinant peptide pieces. Scale bar = 200 μm

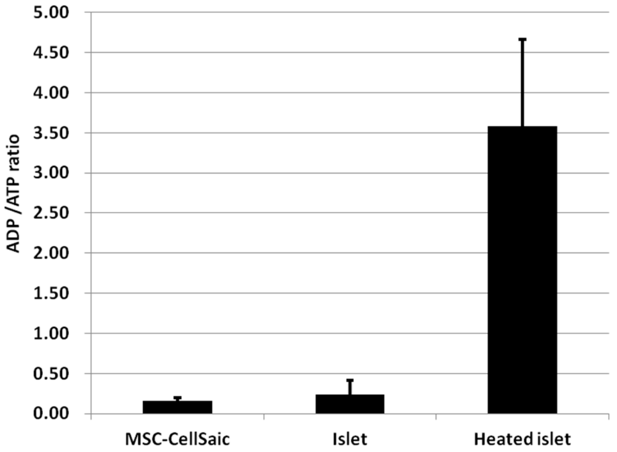

Figure S2. Evaluation of Islet Cell Viability. Islet survival was evaluated by ADP/ATP ratio assay using the ADP/ATP Ratio Assay Kit, ApoSENSOR (K255-200, BioVision, San Francisco, USA). Islet survival was evaluated by ADP/ATP ratio assay using the ADP/ATP Ratio Assay Kit, ApoSENSOR (K255-200, BioVision, San Francisco, USA). As a control for necrotic islets, islets heated at 60°C for

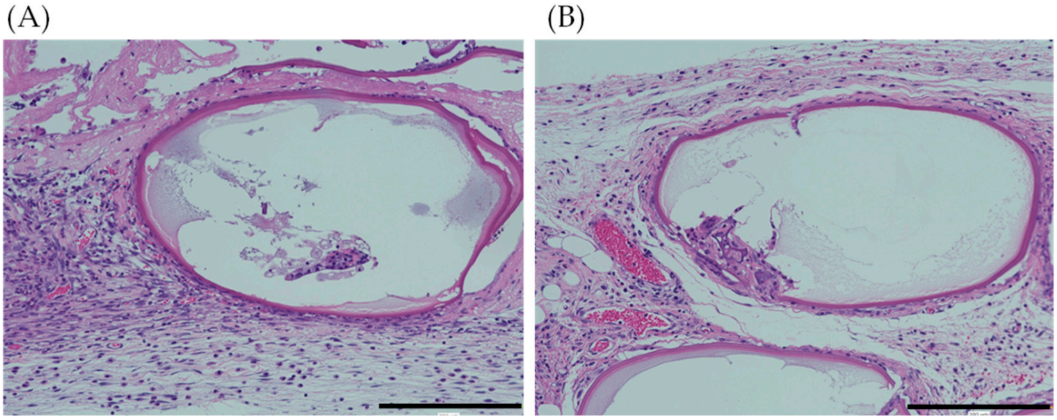

Figure S3. Histological images of microencapsulated islets and MSC-CellSaic at 14 days of transplantation. H&E stained photographs of microencapsulated pancreatic islets (A) and MSC-CellSaic (B) implanted subcutaneously in mice. They were removed 14 days after transplantation and tissue sections were prepared. The inflammatory response around the capsule is mild and the cells inside are viable. scale bar = 200 μm.
